# Polarons in Heterogeneous Photo(electro)Catalysts

**DOI:** 10.1002/anie.202522726

**Published:** 2026-02-25

**Authors:** Hao Wu, Fatwa F. Abdi, Yun Hau Ng

**Affiliations:** ^1^ Macao Institute of Materials Science and Engineering (MIMSE) Faculty of Innovation Engineering Macau University of Science and Technology Taipa Macao SAR China; ^2^ School of Energy and Environment City University of Hong Kong Kowloon Hong Kong SAR China; ^3^ Center For Renewable Energy and Storage Technologies (CREST) Clean Energy Research Platform (CERP) Chemical Engineering Program Physical Science and Engineering (PSE) Division King Abdullah University of Science and Technology (KAUST) Thuwal Saudi Arabia

**Keywords:** charge dynamics, interface reactivity, photo(electro)catalysts, photon absorption, polaron

## Abstract

Heterogeneous photo(electro)catalysis involves sequential steps of photon absorption, charge separation, polaron formation, trapping, bulk and surface recombination, charge extraction, and surface catalysis. Among these, the formation and dynamics of polarons, quasiparticles resulting from strong electron‐lattice interactions, play a pivotal yet often underappreciated role. With ultrafast lifetimes ranging from femtoseconds to picoseconds, polarons are challenging to control, but they crucially influence photon absorption, charge carrier mobility, recombination rates, and catalytic reactivity. Recent advances in time‐resolved spectroscopy, scanning probe microscopy, and theoretical modeling have enabled direct observation and mechanistic interpretation of polaronic states in various photoactive semiconductors. This minireview aims to provide a comprehensive and pedagogical overview of polaron phenomena in heterogeneous photo(electro)catalysts, with a focus on how they affect key material functionalities. Special emphasis is placed on correlating material performance with polaron behavior through state‐of‐the‐art experimental characterization and modeling techniques. By highlighting mechanistic insights and unifying design principles, this minireview aims to guide the rational engineering of semiconductors with tailored polaronic properties for enhanced photo(electro)catalytic performance.

## Graphical abstract

Polarons are defined as quasiparticles consisting of electrons/holes covered by a cloud of virtual phonons, which are beneficial/detrimental to the electronic properties of materials. This minireview provides a conceptual overview of how small polaron formation influences the photon absorption, charge transport and separation, and surface reactivity in heterogeneous photo(electro)catalyts. The state‐of‐the‐art experimental and numerical techniques are also compiled.

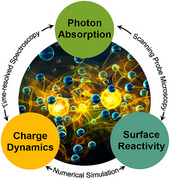

## 1 INTRODUCTION

**FIGURE 1:**
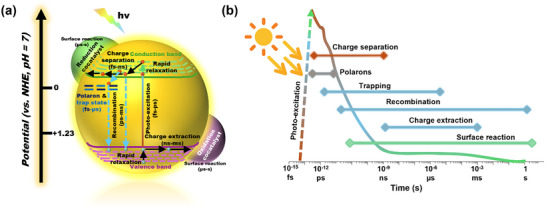
(a) Mechanism of photo(electro)catalytic redox reactions on a heterogeneous photo(electro)catalyst [4]. Adapted and reproduced with permission, Copyright 2022, Royal Society of Chemistry. (b) Timeline of charge‐carrier dynamics in a heterogeneous photo(electro)catalyst from photoexcitation to surface reaction [3]. Adapted and reproduced with permission, Copyright 2021, Nature Portfolio.

Photo(electro)catalysis is a promising approach to harnessing and storing intermittent sunlight as the primary energy source for synthesizing solar fuels [1]. Meanwhile, semiconducting oxides have dominated the research on photo(electro)catalysis owing to their natural abundance, low cost, and relative ease of synthesis [2]. The overall photo(electro)catalytic process involves a series of fundamental steps (Figure 1a). Upon photoexcitation, electrons are promoted from the valence band (VB) to the conduction band (CB), generating charge carriers that are spatially and energetically separated. In many semiconducting oxides, the strong ionic bonding nature leads to pronounced electron‐lattice coupling, favoring the self‐trapping of charge carriers into localized polaronic states [3]. These polarons, characterized by a localized charge and associated lattice distortion, significantly influence the charge carrier transport properties and recombination dynamics of the material. In addition to polaron formation, defect states, such as oxygen vacancies (OVs) and cation interstitials, can act as charge carrier traps, further modifying the recombination pathways and often exacerbating non‐radiative losses. Only the minority of photogenerated carriers that escape trapping and recombination are extracted to the surface to participate in catalytic redox reactions [4], limiting the solar‐to‐chemical conversion efficiency to below 5% in most reported systems [5]. Notably, the surface redox processes typically occur on timescales of milliseconds to seconds, in stark contrast to the ultrafast timescales of polaron formation (sub‐picosecond) and charge extraction (nanosecond), as illustrated in the temporal schematic (Figure 1b). To reconcile these kinetic mismatches, a variety of material engineering strategies have been explored [6]. Importantly, a growing body of evidence suggests that the efficacy of these strategies is intimately linked to their ability to influence polaron characteristics, underscoring the importance of understanding and controlling polaron dynamics as a critical intermediate step linking photon absorption to catalytic reactivity.

Polarons are quasiparticles formed by the coupling of charge carriers with lattice vibrations, creating a localized distortion field often described as a “phonon cloud” [7]. It is important to clarify the conceptual relationship between polarons and the widely discussed notions of defect densities and trap states in semiconductor photo(electro)catalysts. In earlier studies, localized charges were commonly described within a trap‐state framework, where defects or disorder introduce static electronic states inside the bandgap that capture carriers and promote nonradiative recombination [8, 9, 10]. From a modern viewpoint, many of these so‐called trap states can be physically reinterpreted as manifestations of small polarons, in which excess electrons or holes become self‐trapped through strong coupling with local lattice distortions. Unlike a purely electronic trap picture, the polaron framework explicitly incorporates the dynamic electron−phonon interaction, the thermally activated hopping transport, and the continuous evolution between localized and delocalized carrier states. Therefore, polarons are regarded as a more complete physical description that unifies defect chemistry, lattice relaxation, and carrier localization within a single microscopic framework. This perspective also bridges earlier defect‐based interpretations with recent advances in operando spectroscopy and atomistic simulations of charge localization.

**FIGURE 2:**
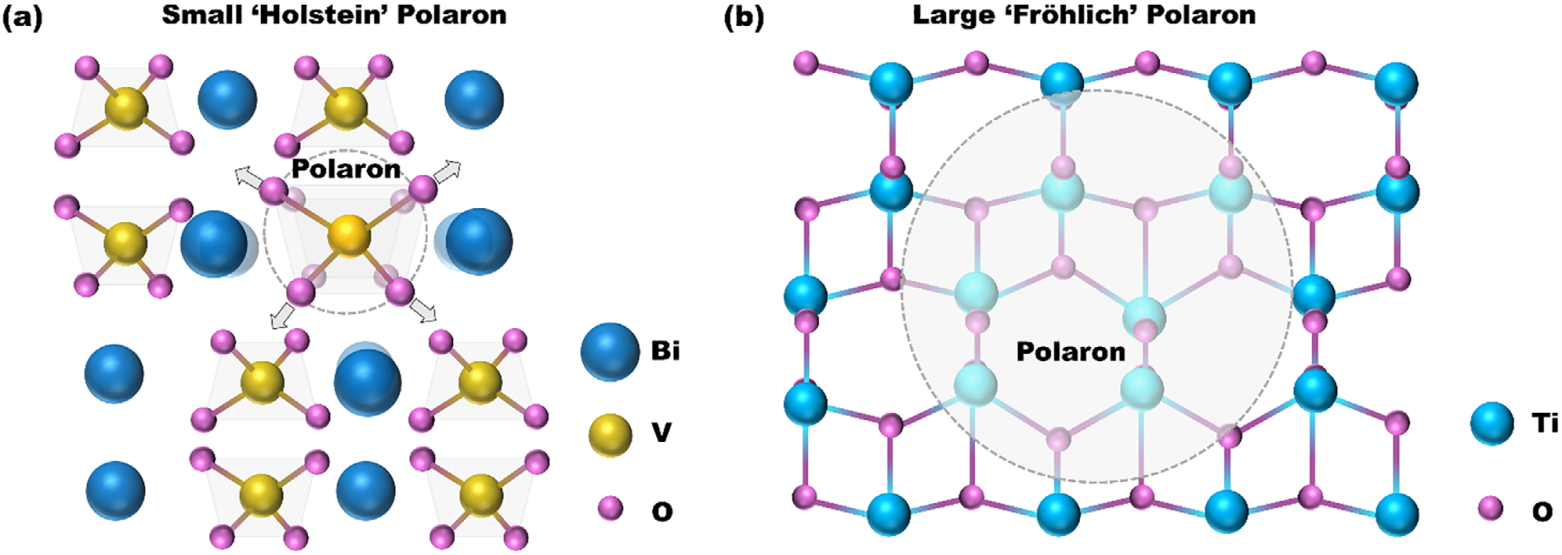
Schematic diagram of (a) the formation of a small polaron (Holstein polaron) at a lattice unit and (b) the formation of a large polaron (Fröhlich polaron) across several lattice units. For illustration, the crystal configurations in (a) and (b) are monoclinic BiVO_4_ and anatase TiO_2_, respectively.

Depending on the spatial extent of the electron‐phonon interaction, polarons are generally categorized into small polarons and large polarons, each exhibiting distinct transport and energetic characteristics. A small polaron (e.g., Holstein‐type) is typically confined within a single lattice site (Figure 2a). It forms a narrow mid‐gap electronic state that lies below the Fermi level (*E_F_*), resulting in a strongly localized charge. Charge transport in this regime occurs via thermally activated phonon‐assisted hopping, which is inherently incoherent and characterized by low mobility, often below 1 cm^2^ V^−1^ s^−1^ [11]. In contrast, a large polaron (e.g., Fröhlich‐type) extends over multiple lattice sites (Figure 2b) and corresponds to a shallow, more delocalized electronic state. Owing to its coherent motion, the large polaron exhibits higher charge mobility (≫1 cm^2^ V^−1^ s^−1^), which notably decreases with increasing temperature, a behavior that contrasts sharply with the small polaron [12]. In addition, polarons can be further categorized as electron polarons or hole polarons depending on the nature of the self‐trapped charge carrier (Table 1), which often correlates with cation‐ or anion‐centered lattice distortions, respectively. These classifications provide a useful framework for understanding how polaron size and type dictate charge mobility, recombination kinetics, and interfacial reactivity in photo(electro)catalytic systems. On one hand, small polarons introduce localized energy states that trap photogenerated carriers and facilitate non‐radiative recombination. On the other hand, under certain conditions, polarons, particularly large ones, can offer beneficial effects. They may enhance charge screening, reduce the scattering of carriers by phonons or defects, and mitigate charge localization at undesirable defect sites [13]. Understanding and manipulating, these contrasting polaronic behaviors is therefore crucial for the rational design of efficient photo(electro)catalysts.

**TABLE 1:** Types of polarons in typical metal oxide photo(electro)catalysts.

Material	Size	Type	Reference
Rutile TiO_2_	Small	Electron	[14]
Anatase TiO_2_	Large	Electron	[15]
SrTiO_3_	Large	Electron	[16]
BaTiO_3_	Small	Electron	[17]
CeO_2_	Small	Electron	[18]
Fe_2_O_3_	Small	Electron	[19]
n‐BiVO_4_	Small	Electron/Hole	[20, 21]
p‐BiVO_4_	Small	Hole	[22]
CuBi_2_O_4_	Small	Electron	[23]

In this minireview, we systematically examine the multifaceted roles of polarons in determining the structure–property‐function relationships of heterogeneous photo(electro)catalysts, with an emphasis on semiconducting oxides. We begin by discussing how polaron formation affects photon absorption and optical transitions. Next, we explore the influence of polarons on charge separation and transport mechanisms, including bulk and surface transport, Fermi level modulation, and interfacial charge transfer, with particular attention to the signatures of small polaron formation. We also highlight recent advances in experimental and theoretical techniques that enable the characterization of polaronic states and their dynamics. Finally, we examine the role of polarons in shaping surface reactivity, especially molecular adsorption and catalytic reactivity. By integrating insights from both fundamental physics and materials chemistry, this minireview aims to provide a conceptual framework and design guidelines for optimizing next‐generation heterogeneous photo(electro)catalysts through polaron engineering.

## 2 Photon Absorption

When a photoactive semiconductor is illuminated with photons whose energy equals or exceeds its bandgap, electrons are excited from the VB to the CB, leaving behind holes. These photogenerated carriers rapidly relax to the band edges, electrons to the CB minimum (CBM), and holes to the VB maximum (VBM), through phonon‐mediated relaxation. Common strategies, including doping [24], defect engineering [25], and quantum confinement [26], have been used to fundamentally tune the band energy positions and subsequently optimize the photon absorption of photoactive semiconductors. These approaches modulate the electronic band structure, typically by introducing sub‐bandgap states that enable lower‐energy transitions involving defect levels or impurity bands. A less discussed, yet fundamentally significant aspect of these modifications is their tendency to induce polarons, localized quasiparticles formed by the coupling of excess charge with lattice distortions. These polarons, arising from intrinsic vacancies or extrinsic dopants, can generate sub‐bandgap absorption features. The formation of polarons not only alters the effective optical bandgap but may also introduce new optical pathways, thereby reshaping the material's photon absorption profile.

**FIGURE 3:**
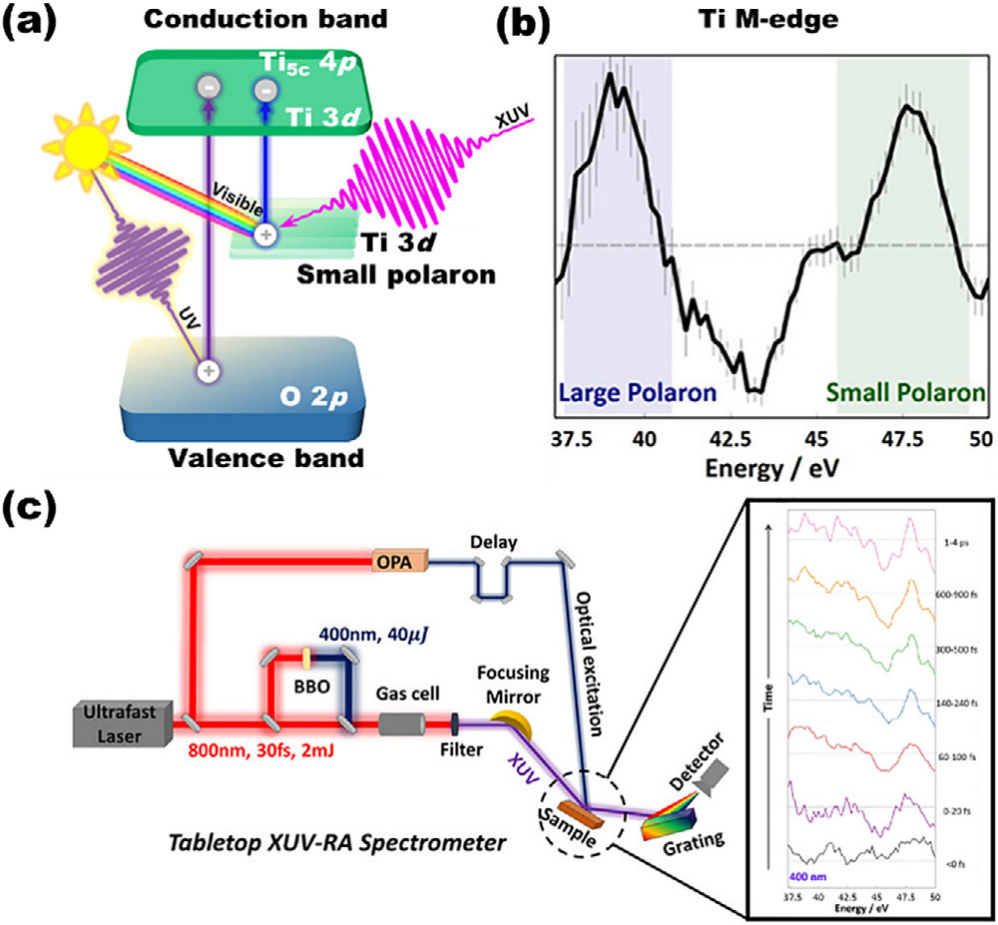
(a) Schematic illustration of band structure for anatase TiO_2_ with electrons excited from the valence band and the small polaron state to the conduction band under UV‐ and visible‐light irradiation. (b) The Ti M_2,3_‐edge XUV‐RA spectrum of anatase TiO_2_. (c) Diagram of the tabletop XUV‐RA spectrometer and the transient Ti M_2,3_‐edge XUV‐RA spectra of anatase TiO_2_ upon 400 nm excitation [15, 27]. Reproduced with permission, Copyright 2022, American Chemical Society.

A representative case is titanium dioxide (TiO_2_), whose photocatalytic activity is largely restricted to the ultraviolet (UV) region, owing to its wide bandgap. Among various defect‐engineering approaches, the introduction of OVs, commonly achieved through hydrogen annealing, has emerged as an effective method to extend the optical response of TiO_2_ into the visible range. The presence of OVs introduces localized excess electrons, which reduce neighboring Ti^4+^ sites to Ti^3+^ and result in the formation of small electron polarons. These defect‐induced polaronic states introduce energy levels within the bandgap and have been correlated with enhanced visible‐light absorption. However, the precise electronic transitions responsible for sub‐bandgap photon absorption in OV‐rich TiO_2_ have long remained elusive. A breakthrough was recently achieved by Baker et al., who employed ultrafast extreme ultraviolet reflection‐absorption (XUV‐RA) spectroscopy, a tabletop high‐harmonic‐generation‐based technique, to directly probe the dynamics of electronic excitation in defective TiO_2_ (Figure 3a) [15, 27]. The XUV‐RA spectra, centered on the Ti M_2,3_‐edge, are uniquely sensitive to the electronic environment of Ti centers and enable differentiation between small and large polaron states (Figure 3b). As previously discussed, small polaron formation induces local lattice expansion due to weakened electrostatic attraction between reduced Ti sites and surrounding O atoms. This structural distortion diminishes the screening of the core‐excited Ti 3p level by neighboring O atoms, resulting in a characteristic blue shift in the XUV absorption features. Upon 400 nm excitation, time‐resolved XUV‐RA measurements captured a transient spectral blueshift consistent with the presence and excitation of small electron polarons (Figure 3c). These observations provide compelling experimental evidence that the visible‐light response of OV‐TiO_2_ arises from the promotion of electrons from localized polaron states into the CB, rather than transitions from the VB to polaron levels, a mechanism that fundamentally redefines the understanding of sub‐bandgap absorption in defective metal oxides.

Beyond enhancing sub‐bandgap absorption, the dynamic formation of small polarons can also lead to a misrepresentation of the optical bandgap in photoactive semiconductors. Cerium dioxide (CeO_2_), a prototypical rare‐earth oxide, is commonly reported to exhibit a bandgap of 3.3–3.6 eV based on Tauc analysis of steady‐state UV–vis spectra [18]. However, recent studies using fast transient absorption spectroscopy (FTAS) have revealed a different picture [28]. Specifically, FTAS measurements suggest a larger intrinsic bandgap of approximately 4.0 eV between the Ce 4f and O 2p states. In the case of CeO_2_, the time‐resolved spectra revealed a pronounced blueshift in the photoinduced absorption (PIA) signal during the first picosecond after excitation, indicating a rapid stabilization of photoexcited electrons via small polaron formation. This stabilization reflects the reduced energy of Ce 4f electrons as they couple with local lattice distortions. Global kinetic analysis of the transient signal further resolved a distinct spectral shift of ∼0.42 eV within the first two picoseconds, attributed to the formation of a small polaron level situated approximately 0.4 eV below the Ce 4f conduction edge, highlighting the importance of incorporating polaron dynamics into the interpretation of light absorption processes in semiconducting oxides.

**FIGURE 4:**
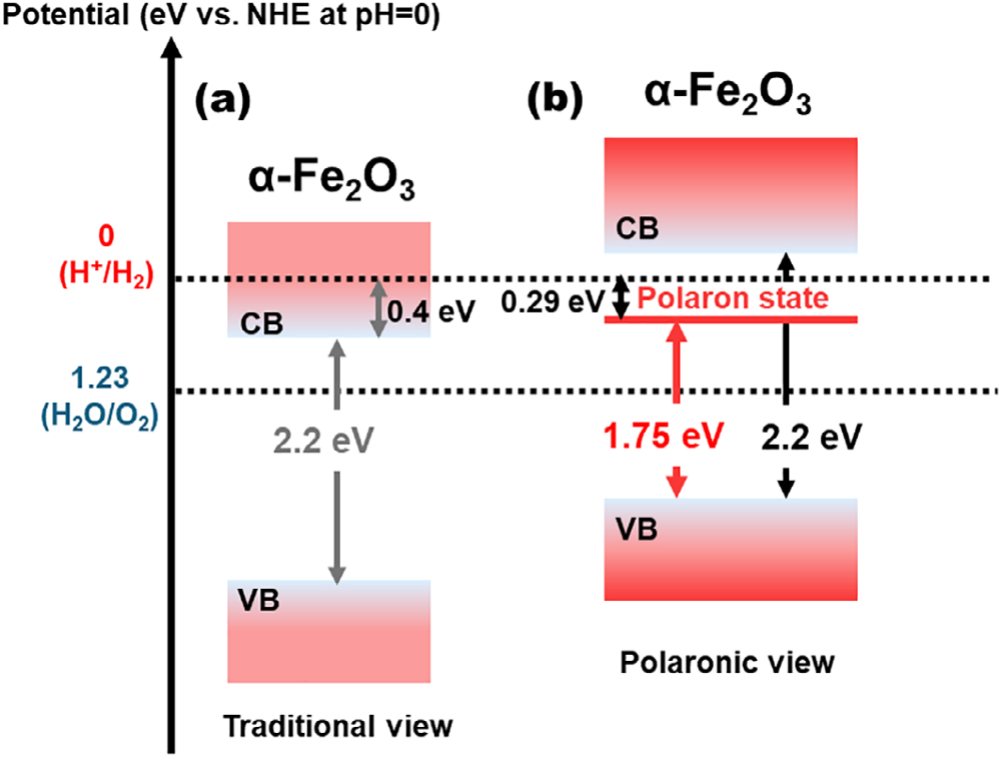
(a) Traditional view and (b) polaronic view of the band edge positions for α‐Fe_2_O_3_ [19]. Adapted and reproduced with permission, Copyright 2018, Nature Portfolio.

Similar polaron‐induced discrepancies have been observed in other metal oxides with strong electron‐lattice coupling. A particularly revealing case is hematite (α‐Fe_2_O_3_), which exhibits an optical bandgap of approximately 2.2 eV, ideally suited for visible‐light harvesting, and possesses a CBM that is theoretically capable of driving proton reduction. However, in practical photo(electro)catalytic water splitting, α‐Fe_2_O_3_ consistently fails to produce hydrogen with any appreciable efficiency. This long‐standing discrepancy has been attributed to ineffective CBM alignment (Figure 4a). Additionally, recent photoelectron spectroscopy (PES) studies by Lohaus et al. revealed that optical excitation in α‐Fe_2_O_3_ induces the formation of an Fe^3+/2+^ small polaron level approximately 0.5 eV below the CBM (Figure 4b) [19]. This newly formed polaron state effectively narrows the accessible polaron‐mediated gap, positioning the occupied electronic states below the redox potential required for proton reduction. As a consequence, although photon absorption may be enhanced via direct excitation into polaronic states, the resulting excited carriers lack sufficient thermodynamic driving force to trigger hydrogen evolution.

## 3 Charge Separation and Transport

**FIGURE 5:**
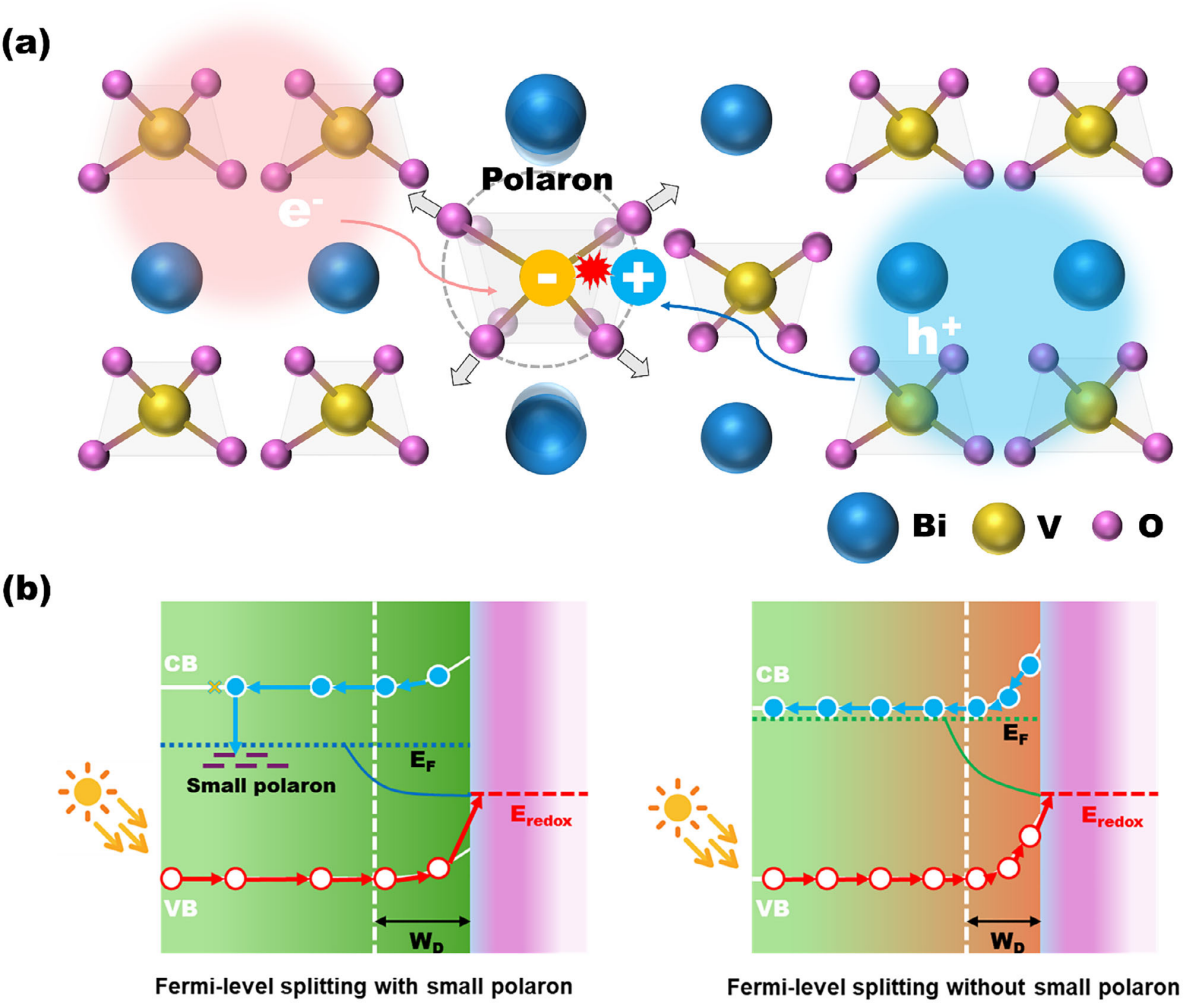
(a) Schematic illustration of the electron self‐trapping (i.e., small electron polaron formation), the hole self‐trapping (i.e., small hole polaron formation), and the sequential electron‐hole recombination [30]. Adapted and reproduced with permission, Copyright 2022, American Chemical Society. (b) Electronic diagrams of Fermi level splitting over a semiconductor photoelectrocatalyst with and without small polaron formation [25]. Small polaron states, depletion width (W_D_), and surface redox potential position (E_redox_) are displayed in the diagrams. Adapted and reproduced with permission, Copyright 2023, American Chemical Society.

Following photon absorption, photoexcited electrons and holes generated deep in the bulk are prone to rapid recombination before reaching the surface [29]. Although intrinsic shallow trap states can temporarily prolong carrier lifetimes, they often do so at the expense of carrier mobility and electronic conductivity [30, 31, 32, 33]. A more fundamental phenomenon influencing this balance is the formation of polarons, particularly small polarons, which arise when a charge carrier (electron or hole) localizes by inducing a local lattice distortion. These small polarons hop between adjacent atomic sites, a thermally activated process that is significantly slower than band‐like transport, thereby reducing effective carrier mobility and aggravating recombination (Figure 5a) [30]. Furthermore, small polarons hinder photoinduced Fermi level splitting, a key parameter that reflects the nonequilibrium potential of charge carriers under illumination (Figure 5b). A limited splitting reduces the photovoltage and ultimately suppresses the driving force for redox reactions. The activation energy (*E*_a_) required for small polaron hopping reflects the energy barrier for lattice rearrangement and strongly impacts the temperature‐dependent conductivity of the material. The polaronic conductivity is typically described by: [34]

(1) $$\sigma \left(T\right)=AT^{-1}exp\left(-E_{a}/kT\right)$$

 where *A* is a pre‐exponential factor, *k* is Boltzmann's constant, and *T* is temperature.

While the discussion of polaron energy levels and densities is often qualitative, representative quantitative ranges can be extracted from both experimental and theoretical studies. For typical oxide semiconductors, the reported small‐polaron binding energies generally fall in the range of ∼0.2–0.8 eV, depending on crystal phase, defect concentration, and surface termination. Correspondingly, polaron densities are closely correlated with dopant or defect concentrations (e.g., OVs), typically spanning 10^16^–10^20^ cm^−3^ under practical synthesis and operating conditions. Experimentally, these quantities are indirectly estimated using techniques such as electron paramagnetic resonance (EPR), optical absorption spectroscopy, transient spectroscopy, and temperature‐dependent conductivity measurements, whereas first‐principles calculations provide complementary access to self‐trapping depths and migration barriers. It should be emphasized that absolute quantification remains challenging due to strong coupling between electronic localization and lattice relaxation, as well as the dynamic nature of polarons under illumination and bias. Nevertheless, even semi‐quantitative comparisons reveal that deeper polaron trapping energies and higher polaron densities generally correlate with reduced mobility but enhanced recombination resistance, thereby exerting a dual influence on charge collection efficiency and interfacial reaction kinetics in photo(electrocatalytic) systems.

A prototypical example of small polaron‐dominated charge transport is found in benchmark metal oxide photo(electro)catalysts such as bismuth vanadate (BiVO_4_), α‐Fe_2_O_3_, and strontium titanate (SrTiO_3_). In these materials, photoexcited electrons commonly migrate via a thermally activated small polaron hopping mechanism, which is orders of magnitude slower than band‐like conduction. In particular, BiVO_4_ suffers from intrinsic OVs, which are difficult to eliminate entirely through either in situ growth control or post‐synthetic treatments. These OVs tend to localize excess electrons at neighboring vanadium (V) sites, reducing V^5+^ to V^4+^ and forming small electron polarons. However, the positively charged OVs electrostatically anchor these polarons, hindering their effective hopping and severely restricting long‐range charge transport in BiVO_4_. To mitigate this issue, a photothermal strategy has been demonstrated to enhance charge mobility by releasing polarons from OV traps [23]. Upon near‐infrared irradiation, a thermally conductive substrate heats the BiVO_4_ photoanode, increasing lattice vibration energy and enabling thermally assisted polaron de‐trapping, which improves photoelectrochemical water oxidation activity. This conclusion was supported by temperature‐dependent intensity modulated photocurrent spectroscopy (IMPS), which showed increased electron diffusion coefficients in BiVO_4_ samples with higher thermal input, attributed to the unbinding of polarons from V^4+^ sites adjacent to OVs. Beyond thermal activation, synthetic modification strategies have also been employed to reduce the density or electronic influence of OVs in BiVO_4_. For instance, BiOIO_3_, a more oxidizing precursor compared to the commonly used BiOI, was used to synthesize BiVO_4_ with suppressed OV formation [35]. This led to a decrease in the bound polaron population, thereby significantly improving charge transport. Similarly, a mild hydrogenation treatment was shown to passivate OVs by inserting hydrogen atoms into vacancy sites. This passivation not only decreased the *E*_a_ for small polaron hopping but also improved overall photoelectrochemical performance by enabling more delocalized electron transport pathways [25]. These findings highlight that precise tuning of polaron‐defect interactions presents a valuable avenue for enhancing the performance of heterogeneous photo(electro)catalysts.

In addition to OVs, the incorporation of external dopants can also mediate polaron transport. Molybdenum (Mo) doping in BiVO_4_ photoanodes reduced the small electron polaron hopping barriers and improved the charge transport [36], as evidenced by the temperature‐dependent conductivity measurements, which show a decreased *E_a_* from 557 meV for undoped BiVO_4_ to 382 meV. Density functional theory (DFT) calculations using the climbing nudged elastic band (cNEB) method also supports the smaller hopping barrier of the electron polaron in Mo‐doped BiVO_4_, which is mainly attributed to the larger volume of Mo^6+^O_4_ than V^5+^O_4_, minimizing the local structural expansion and the corresponding small electron polaron formation. Besides, non‐metallic phosphorus dopants also enable an efficient low‐bias photoelectrochemical water oxidation of BiVO_4_ photoanode via mediating small electron polaron hopping [37]. Similarly, temperature‐dependent electrochemical impedance spectroscopy (EIS) measurements and cNEB simulations evidence the reduced small electron polaron hopping barriers of BiVO_4_ after the phosphorus incorporation. Moreover, the small electron polaron binding energy state was uplifted from 0.39 eV for the pristine BiVO_4_ to 0.34 eV upon incorporating phosphorus. It suggests that the mediated small electron polaron energy level became less stable, and the confined electrons could be more easily re‐excited to the delocalized CB by photons, enhancing charge delocalization and reducing recombination loss. It is noteworthy, however, that a systematic discrepancy exists between experimentally derived *E*_a_ and those calculated via DFT approaches, with experimental values often being significantly higher. This divergence likely stems from the inherent complexity of real materials, including the incomplete quantification or uncontrolled distribution of intrinsic defects such as OVs, as well as the additional Coulombic interactions arising from both positive and negative polaronic screening. These many‐body effects, often neglected or approximated in standard DFT, highlight the need for more advanced computational treatments that incorporate explicit electron–phonon coupling, polaron‐defect interactions, and temperature‐dependent disorder to better replicate experimental observations.

Recent studies have also demonstrated that hydrated transition metal ions in solution can serve as dynamic mediators to facilitate small electron polaron transport and interfacial charge transfer. A particularly compelling example involves the use of Fe(III) ions in a BiVO_4_‐based photocatalytic system for the degradation of sulfamethoxazole, where the introduction of Fe^3+^ boosted the photocatalytic activity by a remarkable 684‐fold compared to the Fe‐free system [38]. The key to this enhancement lies in the redox cycling between Fe(III)/Fe(IV), which couples with the dissociation of small electron polarons in BiVO_4_. Upon light irradiation, the small electron polaron is destabilized and dissociates, enabling the reduction of Fe(III) to transient Fe(II) and the oxidation of Fe(III) to reactive Fe(IV) species. The latter possesses an exceptionally low kinetic barrier (5.4 kJ mol^−1^) for oxygen atom transfer, thus accelerating oxidative degradation reactions at the solid–liquid interface. From a mechanistic standpoint, this study exemplifies how charge‐compensated polaron dynamics, facilitated via redox‐active ionic species, can provide a viable route to overcome intrinsic mobility bottlenecks. It also hints at the possibility of engineering interfacial chemical environments to regulate small polaron populations in real time.

Beyond the extensively studied small electron polarons, small hole polarons are also prevalent in semiconducting oxides, where they play a distinct and often advantageous role in facilitating photo(electro)catalytic water oxidation. Compared with their electron counterparts, small hole polarons generally possess lower binding energies and smaller self‐trapping barriers, allowing more facile transport under ambient conditions. In BiVO_4_, for example, the *E*_a_ for hole polaron hopping has been experimentally determined to be as low as ∼90 meV, based on time‐resolved THz conductivity spectroscopy (TRTC) measurements [21]. This exceptionally low barrier implies that hole transport is not strongly limited by lattice distortion, enabling more efficient migration to catalytic surface sites. From a theoretical perspective, hybrid DFT calculations also suggest that small hole polarons in BiVO_4_ exhibit low self‐trapping energies, such that at finite temperatures, they can be thermally deconfined and behave more like delocalized valence band carriers. This implies that hole polarons in BiVO_4_ may serve as “quasi‐free” holes under realistic operating conditions, enabling sustained oxidation activity with reduced recombination losses [22]. Moreover, while small electron polarons typically localize on V sites, small hole polarons preferentially form at bismuth (Bi) sites, inducing local lattice contraction characterized by a shortening of the Bi─O bond from 2.47 to 2.42 Å. This site selectivity underscores the anisotropic nature of polaron formation in multicationic oxides, where the specific orbital character and bond covalency govern polaron localization.

**FIGURE 6:**
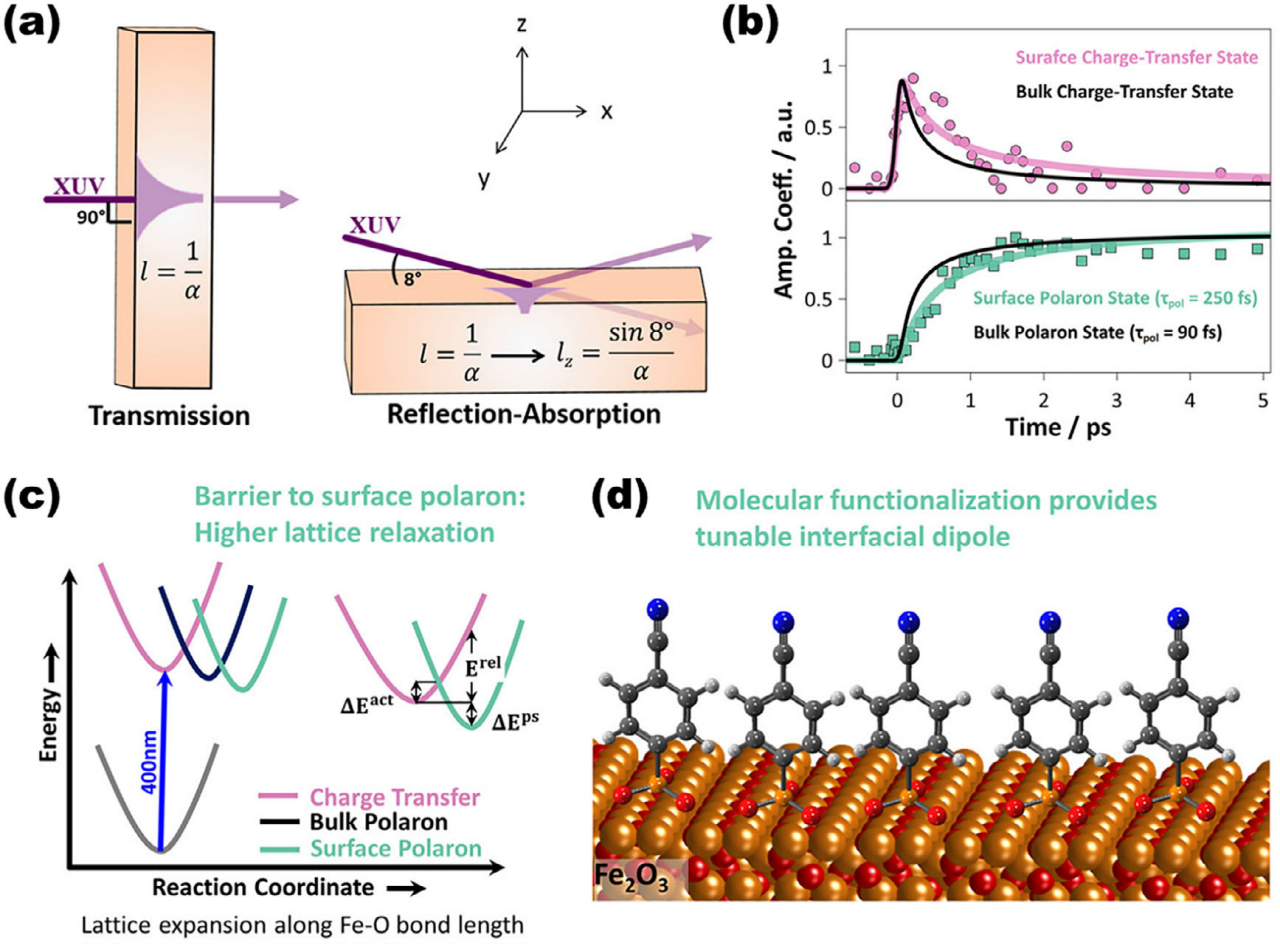
(a) Schematic illustration of the time‐resolved XUV spectrometer in the transmission and reflection‐absorption geometry [39]. Reproduced with permission, Copyright 2018, Royal Society of Chemistry. (b) Time‐resolved XUV reflection‐absorption spectra with surface and bulk charge‐transfer state and surface and bulk polaron state for α‐Fe_2_O_3_. (c) Marcus model of charge transfer and bulk and surface polaron formations along the Fe─O reaction coordinate. Δ*E* ^act^, Δ*E* ^rel^, and Δ*E* ^ps^ represent polaron activation energy, relaxation energy, and stabilization energy, respectively. (d) Schematic illustration of molecular functionalized α‐Fe_2_O_3_ surfaces [40]. Reproduced with permission, Copyright 2019, AIP Publishing.

The energetics and dynamics of polaron formation at surfaces are markedly distinct from those in the bulk, owing to structural, electronic, and environmental asymmetries at the solid–liquid interface. Recent advances in surface‐sensitive spectroscopy, particularly XUV‐RA techniques operated under near‐grazing incidence, have provided direct insights into these differences (Figure 6a) [39]. In photoexcited α‐Fe_2_O_3_, the rate of surface polaron formation was found to be approximately three times slower than that of bulk polarons, as revealed by time‐resolved XUV‐RA measurements (Figure 6b,c) [40]. This observation suggests a stronger polaron binding energy at the surface, likely arising from enhanced lattice flexibility near the interface. The reduced steric constraints at the surface compared to the bulk allow for more significant lattice expansion, which stabilizes the self‐trapped carrier state and slows down its formation dynamics. These insights also highlight the role of steric effects in governing polaron behavior, opening up avenues for interface engineering. For instance, surface modification with self‐assembled monolayers or ligands that impose steric hindrance could potentially suppress lattice relaxation, thereby lowering the polaron binding energy and facilitating more efficient surface charge transfer (Figure 6d). Interestingly, these experimental findings contrast with theoretical predictions from DFT, which often suggest that surface polaron states are less stable than those in the bulk, even in the presence of explicit water layers or surface termination models [41, 42]. This discrepancy is likely attributed to the ubiquitous presence of OVs on real catalyst surfaces, which are often underrepresented or oversimplified in DFT models. Notably, a recent DFT study on the (010) surface of BiVO_4_ revealed that surface OVs significantly enhance polaron binding, making the surface state more energetically stable than the pristine or bulk counterparts [43]. These stabilized surface polarons can act as non‐radiative recombination centers, thereby compromising charge separation and limiting overall photoactivity.

**FIGURE 7:**
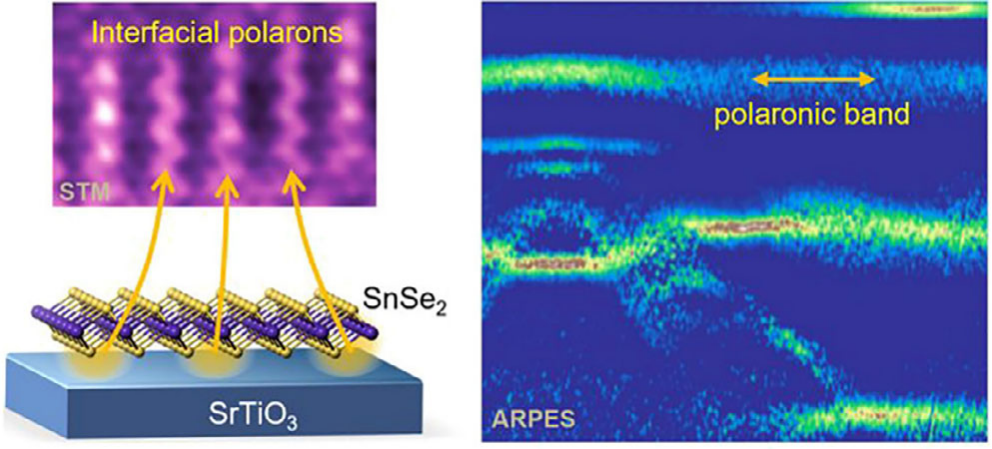
Schematic illustration of the geometric structure and ARPES spectra of the monolayered SnSe_2_/STO sample [44]. Reproduced with permission, Copyright 2020, American Chemical Society.

Although the polaron formation at the surface and in the bulk has recently been investigated in metal oxide photo(electro)catalysts, the polaron's effect on the interfacial charge transfer is still much under‐explored, which is meaningful to understand the underlying mechanism that promotes the charge separation of heterostructured photo(electro)catalyst composites. Combining angle‐resolved photoemission spectroscopy (ARPES) and low‐temperature scanning tunneling microscopy (STM) enables the study of interfacial polarons in SnSe_2_/Nb‐doped SrTiO_3_ (Figure 7) [44]. The ARPES spectra of SnSe_2_/Nb‐doped SrTiO_3_ show an isolated band at approximately ‐0.7 eV in the middle of the gap. The STM data of the SnSe_2_ monolayer and the APRES spectra of SrTiO_3_ exclude the possibility of impurity states in SnSe_2_ and SrTiO_3_, respectively. The interfacial electron transfer from Nb‐doped SrTiO_3_ to SnSe monolayer induces a surface structural rearrangement in the underly Nb‐doped SrTiO_3_ surface, resulting in the formation of interfacial polarons. The observed deep in‐gap band suggests its small polaron nature, which potentially results in decreased interfacial charge separation efficiency. This observation also underscores the need to treat polarons not only as bulk or surface phenomena, but as interfacial quasi‐particles whose presence and behavior are strongly modulated by band alignment, strain, electrostatics, and local dielectric screening at the heterojunction.

**FIGURE 8:**
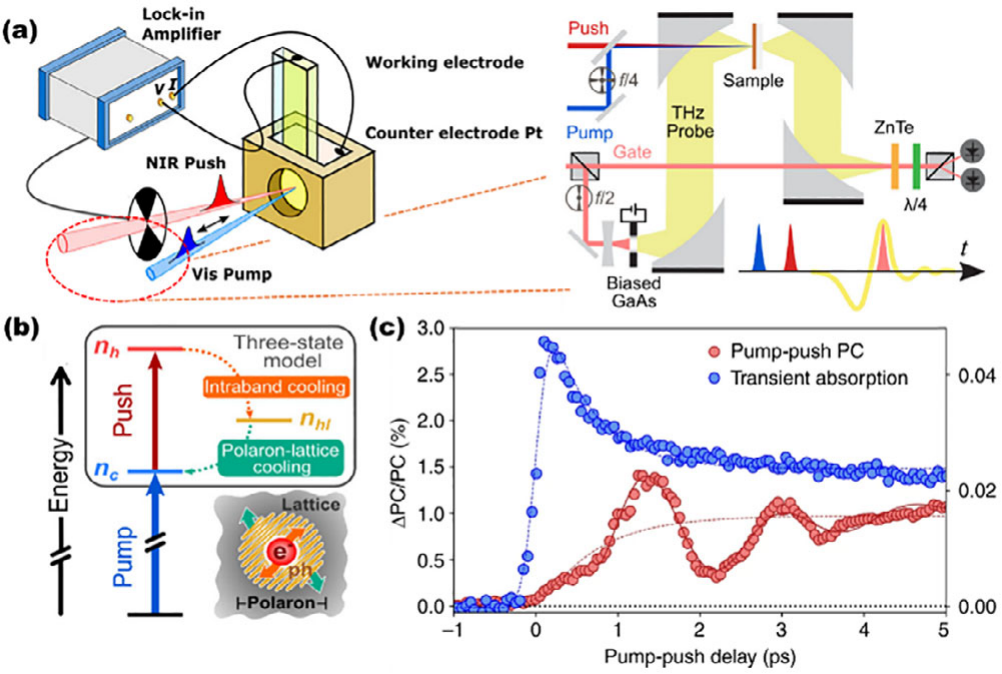
(a) Schematic illustration of the pump‐push‐photocurrent detection setup for photoelectrochemical devices. (b) Schematic energy diagram of the polaron model to interpret pump‐push‐photocurrent [46, 47]. Adapted and reproduced with permission, Copyright 2021 and 2023, American Chemical Society. (c) Transient absorption decays and pump‐push‐photocurrent curves of α‐Fe_2_O_3_ [45]. Reproduced with permission, Copyright 2019, Nature Portfolio.

Most experimental investigations of polaron formation in metal oxide photo(electro)catalysts are performed under ex situ or vacuum‐based conditions, which deviate significantly from the actual operating environments of photocatalytic suspensions or photoelectrochemical cells. This methodological limitation introduces uncertainty in correlating observed polaron characteristics with their actual roles in charge separation and catalytic reactivity. Therefore, in situ or *operando* characterization techniques capable of probing polaron dynamics under realistic working conditions are highly desirable yet remain technically challenging and relatively underdeveloped. A pioneering effort by Bakulin et al. employed pump‐push‐photocurrent spectroscopy (PPP) with photocurrent readout to investigate electron polaron formation in α‐Fe_2_O_3_‐based photoelectrochemical cells under operational bias and illumination (Figure 8a,b) [45]. Their work revealed the ultrafast formation of Fe(II) electron polarons within ∼600 fs (Figure 8c) and estimated the corresponding reorganization energy to be ‐0.5 eV. This technique bridges ultrafast carrier dynamics with macroscopic current response, providing real‐time insights into the electronic relaxation pathways relevant to charge extraction. Interestingly, a comparison with extreme ultraviolet (XUV) transient absorption spectra centered at the Fe M_2,3_ edge for solid‐state α‐Fe_2_O_3_ (under dry and ex situ conditions) suggests an even faster sub‐100 fs localization and a slightly smaller polaron stabilization energy of ‐0.4 ± 0.05 eV. The contrast in timescales and energetics between the in situ and ex situ measurements implies that water molecules and electrolyte species in the photoelectrochemical environment likely stabilize the polaronic state, possibly through hydrogen bonding or dipole‐field interactions at the semiconductor‐electrolyte interface. These findings highlight that the surrounding chemical environment can exert a profound influence on polaron formation energetics and dynamics, and that *operando* spectroscopies are essential to revealing the true nature of polarons under functional photo(electro)catalytic conditions.

## 4 Surface Chemical Reactions

Photo(electro)catalysis ultimately hinges on the interfacial redox reactions with adsorbed species occurring at the solid–liquid boundary. The kinetics and thermodynamics of these surface reactions are largely governed by the binding strength of adsorbates and the activation barriers of bond‐breaking or bond‐forming steps, both of which are strongly influenced by the filling of antibonding orbitals and the degree of orbital hybridization between the adsorbate and surface atoms. Experimental and theoretical insights have pointed to the potentially profound role of polaron‐adsorbate interactions in modulating surface reactivity. Specifically, the presence of small polarons at the surface may create new surface reactive sites via local lattice distortion and symmetry breaking, modify the local charge distribution and charge transfer kinetics through polaron‐assisted hopping pathways, and tune the surface energy landscape, affecting adsorption and desorption thermodynamics.

**FIGURE 9:**
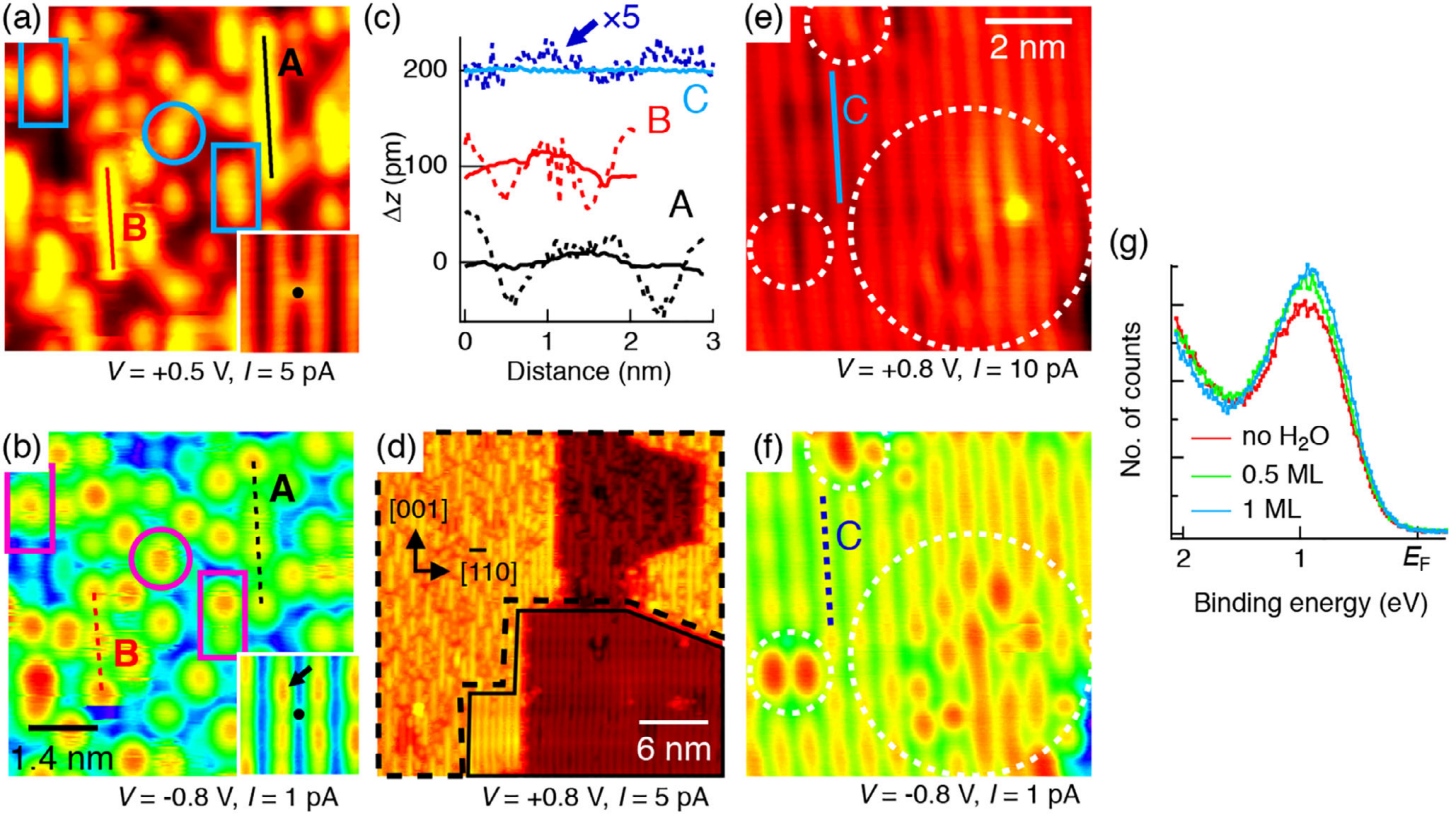
(a) Empty and (b) filled state (dual mode) STM images of the (110) crystal of rutile TiO_2_. (c) The corresponding line profiles taken along two strands in (a and b) and those taken along one section of the continuous water chain in (e and f). (d) Empty state STM image taken from the surface in (a) after annealing to 146 K. (e and f) Dual mode STM images of the perfect region of the (110) crystal of rutile TiO_2_. (g) UPS spectra of rutile TiO_2_ with varied water adsorptions at 200 K [14]. Reproduced with permission, Copyright 2018, American Chemical Society.

A particularly illustrative example of polaron‐adsorbate interaction is the modulation of polaron distribution between surface and subsurface regions, which has a profound influence on surface chemical reactivity. STM and ultraviolet photoelectron spectroscopy (UPS), in conjunction with DFT calculations, have demonstrated that excess electrons in TiO_2_ can form localized polarons that are preferentially stabilized at the topmost surface layer upon adsorption of water or methanol molecules (Figure 9a–g) [14]. This phenomenon is attributed to strong polaron‐adsorbate coupling, wherein the adsorbates act as surface anchors, energetically attracting and localizing electron polarons at reactive sites. Such adsorbate‐induced surface affinity to polarons not only influences their spatial localization but also has significant implications for surface redox chemistry. The surface‐trapped polarons can enhance the likelihood of interfacial electron transfer by increasing their overlap with adsorbed reaction intermediates, thus improving catalytic performance. This mechanism suggests a promising strategy to modulate surface reactivity by selectively engineering the adsorbate environment to direct polaron localization. Complementary theoretical studies employing first‐principles molecular dynamics (FPMD) and nonadiabatic molecular dynamics (NAMD) further support this picture [48]. These simulations reveal that water molecules on the TiO_2_ surface can strongly couple with electron polarons. Moreover, when water dissociates into hydrogen and hydroxyl species on the surface, first‐principles calculations show that the resulting surface configuration can decouple the localized electron polaron from the hydrogen atom, facilitating proton transfer and diffusion across the surface [49]. This insight points to a previously underappreciated dynamic role of polaron‐adsorbate interactions, not only in regulating electronic properties but also in influencing ionic mobility.

**FIGURE 10:**
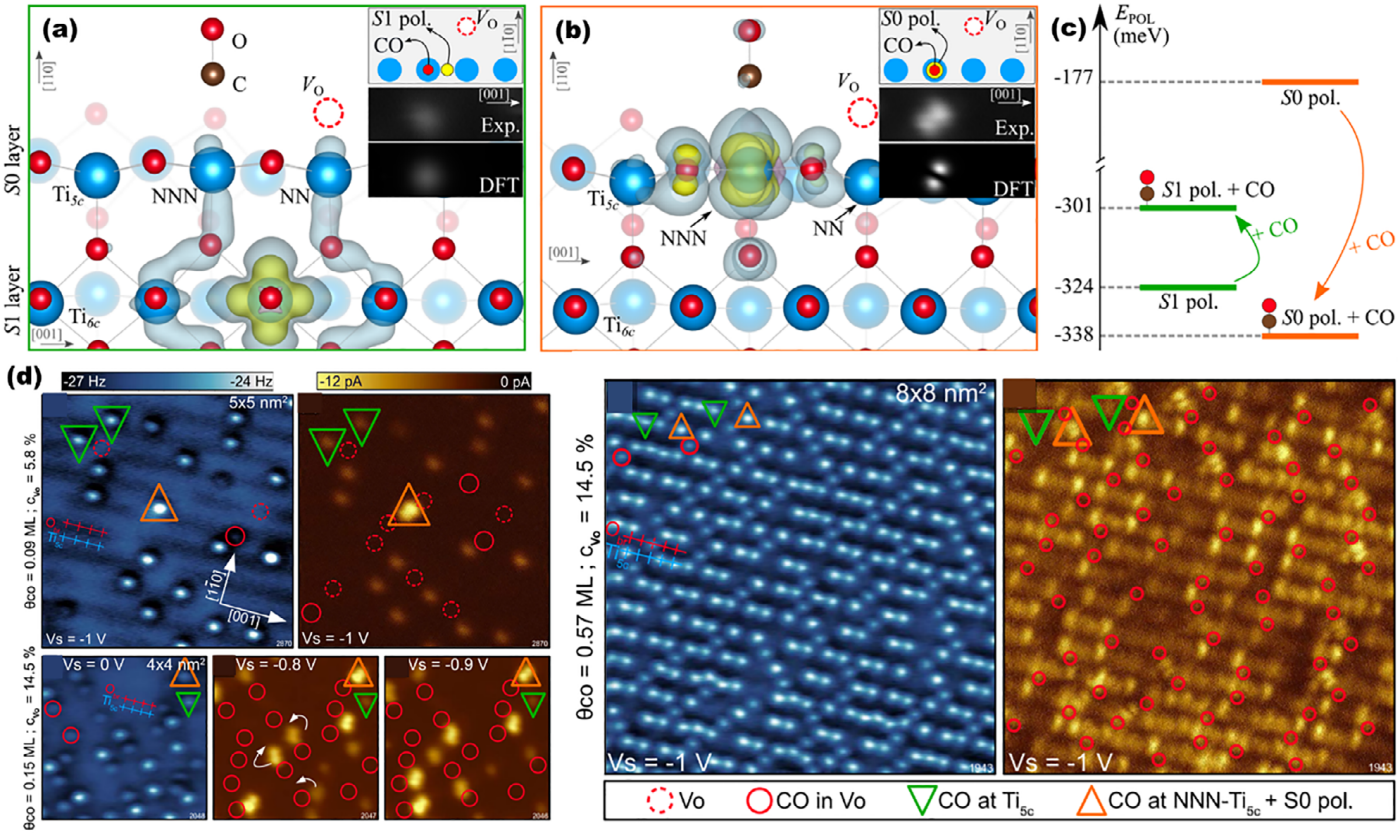
Electronic charge density of (a) the subsurface layer (S1) and (b) the surface layer (S0) polarons in presence of CO. (c) Polaron formation energy of S0 and S1 polarons of the (110) crystal of rutile TiO_2_ with and without adsorbed CO. (d) Experimental constant‐height nc‐AFM (blue‐white) and filled‐state STM images (yellow‐black) of CO adsorbed on the (110) crystal surfaces of rutile TiO_2_ at different surface reduction states [50]. Reproduced with permission, Copyright 2019, American Physical Society.

Beyond water molecules, nonpolar adsorbates can also modulate the spatial distribution and electronic characteristics of surface polarons. A notable example involves carbon monoxide (CO) adsorption on rutile TiO_2_(110) surfaces. Combining non‐contact atomic force microscopy (nc‐AFM) and STM with DFT calculations, researchers have shown that CO can induce subsurface‐to‐surface polaron migration due to favorable polaron‐CO coupling interactions (Figure 10a–d) [50]. This behavior provides further evidence that surface adsorbates can dynamically reshape the polaronic landscape of oxide surfaces and influence their photo(electro)catalytic activity. Specifically, the CO adsorbed on a rutile TiO_2_(110) surface is identified by three different adsorption configurations of CO at OV sites, CO at Ti_5c_ weakly interacted with polarons in the subsurface and strongly interacted CO with the top surface layer polaron at the Ti_5c_ site next nearest neighbor to the OV (NNN‐Ti_5c_). Among them, the third configuration demonstrated the strongest polaron‐CO interaction, leading to enhanced electronic localization at the surface and potentially higher reactivity of the Ti_5c_ site. This effect is especially significant because Ti_5c_ sites are known to act as primary active centers in many photocatalytic reactions, including CO oxidation and water splitting. The promotion of polaron diffusion toward catalytically relevant sites by CO adsorption also implies that surface coverage by reactive molecules can act as a dynamic electronic modifier, shaping both the energetics and kinetics of surface redox processes. In analogy to the case of water‐induced proton mobility, this CO example underscores a broader concept that adsorbate‐driven polaron redistribution is a tunable handle to enhance or suppress local catalytic activity in heterogeneous systems.

While most insights into the polaron‐adsorbate interaction in ternary oxides have traditionally been derived from numerical simulations [51], recent advances in experimental techniques have enabled more direct investigations under relevant catalytic conditions. A notable example is the application of ambient‐pressure resonant photoemission spectroscopy (AP‐ResPES) to probe the surface electronic structure of Mo‐doped BiVO_4_(010) in both ultra‐high vacuum (UHV) and under 0.05 Torr water vapor pressure [52]. The AP‐ResPES measurements reveal a pronounced enhancement of the spectral intensity near the VB edge upon exposure to water molecules. When interpreted in conjunction with DFT calculations, this enhancement is attributed to the formation of small electron polarons, which emerge through interactions with dissociated water molecules, specifically, hydroxyl groups that bind at the VO_4_ sites on the BiVO_4_ surface. This experimental evidence provides compelling support for polaron‐adsorbate coupling under quasi‐operational conditions. The observed spectral changes suggest that adsorbed hydroxyl groups participate in water oxidation and stabilize surface polarons, effectively modulating the local electronic structure and potentially altering the energetics of subsequent surface redox reactions. The exploration of adsorbate‐polaron interactions represents a critical frontier in understanding the surface chemical reactivity of heterogeneous photo(electro)catalysts.

Beyond tuning adsorbate binding energies, surface‐localized polarons can actively generate and expose catalytically active sites through lattice distortion, defect stabilization, and redox‐state modulation. For instance, electron polarons in oxide photoelectrodes are known to stabilize reduced metal centers (e.g., V^4+^ in BiVO_4_ or Ti^3+^ in TiO_2_), thereby creating highly reactive adsorption sites for key intermediates such as *OH and *OOH. Simultaneously, polaron‐induced lattice relaxation can lower the formation energy of OVs, further increasing the density of catalytically accessible sites. Importantly, these microscopic polaron behaviors are directly manifested in macroscopic photo(electro)catalytic metrics. Taking the photoelectrochemical water oxidation as an example, a lower polaron migration barrier facilitates charge delivery to surface active sites, leading to enhanced photocurrent density, while excessive polaron binding can deepen trap states and shift the onset potential anodically. Likewise, the interfacial lifetime of polarons governs the competition between surface reaction and bulk recombination, thereby impacting catalytic turnover and operational stability. Establishing such quantitative links between polaron energetics, surface reactivity, and photo(electro)catalytic performance is essential for translating polaron physics into rational catalyst design principles.

## 5 Summary and Outlook

Although the study of polaron physics began decades ago, it is only recently that their multifaceted roles in photo(electro)catalysis have begun to be systematically understood and exploited. In this minireview, we have highlighted how polarons govern the overall performance of heterogeneous photo(electro)catalysts.

1. The formation of localized polaron states imposes intrinsic limitations on the splitting of the quasi‐Fermi levels, which consequently affects the driving force for redox reactions. Therefore, a full evaluation of the energetic positions of both electron and hole polaron states is necessary to determine the effective bandgap for photo(electro)catalysis. This polaron‐defined bandgap can diverge significantly from the conventional optical bandgap and may serve to reconcile discrepancies frequently observed between experimental measurements and theoretical predictions.
2. Extensive studies have been devoted to correlating the energetics and kinetics of polaron formation in the bulk and on the surface with charge transport and separation. Besides, the emerging recognition of interfacial polarons opens a promising new direction. These interface‐confined polarons likely play a critical role in mediating interfacial charge transfer, and future studies should seek to unravel the underlying mechanisms linking interfacial polaron dynamics with overall photo(electro)catalytic activity. Such mechanistic insights may help decode long‐standing challenges in heterostructured systems, such as charge accumulation, interfacial recombination, and band alignment tuning.
3. The polaron‐adsorbate interaction can significantly modulate not only the localization and mobility of surface/subsurface polarons but also the configuration, bonding, and activation energy of the adsorbed species. While current investigations are largely limited to prototypical molecules such as water and CO, expanding the study to more catalytically relevant species, such as CO_2_, N_2_, NO_3_^−^, and NO_2_^−^, is both necessary and timely [53, 54, 55]. Along this direction, in addition to the commonly applied experimental and theoretical approaches, visualizing the adsorbate‐induced polaron behavior on the surface of heterogeneous photo(electro)catalysts is very meaningful.

In addition to outlining the conceptual framework of polarons in heterogeneous photo(electro)catalysis, this minireview has also surveyed a range of state‐of‐the‐art experimental and theoretical techniques that enable the probing of polaron‐related phenomena. Notably, the distinct thermally activated behaviors of small and large polarons make temperature a primary control variable in probing polaron dynamics. In this context, temperature‐dependent spectroscopies, such as EIS and IMPS, and transient absorption spectroscopy, have proven particularly valuable for unraveling the kinetics of polaron formation and transport [56]. It is important to emphasize that most of the characterization techniques discussed above are inherently macroscopic in nature. As such, the extracted polaronic properties often represent ensemble‐averaged behaviors across the bulk sample, which may obscure critical local heterogeneities intrinsic to realistic photo(electro)catalysts. However, polaron formation, stabilization, and transport are highly sensitive to local structural and electronic environments, especially in heterostructured or defect‐rich systems. These environments include variations in surface crystallinity, atomic coordination geometry, defect distributions, and interfacial lattice mismatch, all of which can locally modulate the strength, dynamics, and mobility of small polarons. To gain a more complete and representative understanding of polaron physics in heterogeneous photo(electro)catalysts, we propose the use of combinatorial measurement approaches that integrate techniques with high selectivity, temporal resolution, and spatial resolution. Such an approach would allow the mapping of polaron behaviors in microscopically distinct regions, bridging the gap between local structure and macroscopic function. Promising combinatorial strategies include temperature‐dependent IMPS for extracting bulk and interface charge dynamics, the integration of time‐resolved spectroscopies such as PPP spectroscopy for temporally resolved insights under *operando* conditions, and scanning probe techniques like low‐temperature STM and nc‐AFM, which can directly visualize polaron‐induced local lattice distortions and electronic states with nanometer spatial resolution. By correlating local spectroscopic signals with *operando* charge transport measurements, such multimodal and spatially resolved characterizations will be critical to uncovering the nuanced roles of polarons in charge separation and transfer processes [57, 58, 59].

Polaron physics is poised to evolve from a passive descriptor of charge localization into an active design parameter for photo(electro)catalyst engineering. Key polaron characteristics, including binding energy, migration barrier, and interfacial lifetime, can now be intentionally tuned through lattice‐softness control, defect and dopant engineering, and heterointerface modulation. For instance, introducing moderate lattice compliance enables stabilization of mobile large polarons that support long‐range charge transport while avoiding deep self‐trapping. At the device level, rational polaron engineering offers a powerful route to simultaneously enhance efficiency and stability. Optimizing polaron mobility accelerates charge extraction and boosts photocurrent, whereas suppressing excessively deep polaron traps mitigates recombination losses and retards photocorrosion. Meanwhile, stabilizing surface‐localized polarons can prolong the lifetime of catalytically active redox sites under operation. These insights suggest that polaron‐centered materials design will become an essential paradigm for developing next‐generation photo(electro)catalysts with high efficiency and long‐term durability. The intersection of polaron physics with heterogeneous photo(electro)catalysis offers a rich and still largely untapped landscape for fundamental discovery and technological innovation. A deeper mechanistic understanding of polaron dynamics and their interactions with real reaction environments will not only reshape our conceptual framework but also guide the rational design of next‐generation heterogeneous photo(electro)catalysts.

## Conflicts of Interest

The authors declare no conflicts of interest.

## Data Availability

Data sharing is not applicable to this article as no new data were created or analyzed in this study.
